# Do Primary Care Physicians Contribute to the Immunization Status of Their Adult Patients? A Story of Patients' Overconfidence Coupled With Physicians' Passivity

**DOI:** 10.3389/fmed.2021.655734

**Published:** 2021-06-17

**Authors:** Thibaut Papis, Christine Clavien

**Affiliations:** Département de santé et médecine communautaires, Institut Ethique Histoire Humanités, Université de Genève, Genève, Switzerland

**Keywords:** adult patient, complacency, false belief, immunization status, overconfidence, perceptual bias, primary care physician, vaccination

## Abstract

**Context:** Immunization coverage counts among the priorities of public health services. To identify factors that motivate or fail to motivate patients to update their vaccination status would help to design future strategies and awareness campaigns.

**Objective:** Our aim was to assess the impact of primary care physicians on the immunization status of their adult patients, and to explore possible explanations.

**Methods:** We invited students and collaborators of Geneva University to bring their paper vaccination records to receive an assessment of their immunization status and personalized vaccination recommendations. Participants completed a first questionnaire at the recruitment phase, and a second 2–3 months later. We assessed their immunization status with the viavac algorithms based on the Swiss national immunization plan.

**Results:** Having a primary care physician did not correlate with better immunization status: only 22.5% patients who reported having a physician and 20% who reported having no physician were up-to-date (*n* = 432; *p* > 0.5). A linear regression indicates that the frequency of medical consultations did not affect patients' immunization status either. Even the participants who recently showed their vaccination record to their primary care physician did not have a better vaccination status. We explored possible explanatory factors and found evidence for the patients' overconfidence about their own immunization status: 71.2% of the participants who predicted that they were up-to-date were wrong about their actual status, and 2–3 months after having received their immunization assessment, 52.8% of the participants who “remembered” having received the assessment that they were up-to-date were wrong: they had in fact received the opposite information that they were not up-to-date. This substantial proportion of wrong beliefs suggests that adult patients are unworried and overconfident about their own immunization status, which is likely to induce a passive resistance toward vaccination updating.

**Conclusions:** This study indicates that the vaccination coverage and beliefs of adults about their immunization status is suboptimal, and that primary care physicians need further support to improve their health-protection mandate through routine immunization check-ups. We highlight that the current covid vaccination campaigns offer a rare opportunity to update patients' immunization status and urge physicians to do so.

## Introduction

Immunization is a key feature of population health (1). In Switzerland, vaccination is not mandatory but the Federal Office of Public Health makes clear recommendations based on updated scientific findings (2). As is the case in most countries across the world (3, 4), the current vaccination coverage of Swiss citizens still needs to be improved; it varies depending on infectious diseases, and it is much higher in children than in older age categories (2). The Covid-19 pandemic increases the importance of vaccination against diseases that may have similar symptoms (5). To convince citizens to maintain their immunization status up-to-date, a combination of strategies is needed, involving primary care medicine, school health services, awareness campaigns, and other initiatives (6–8).

The *Projet Vaccins* was set up in 2018, in collaboration with the Foundation myvaccines (9), with the aim to promote vaccination coverage on the campus of the University of Geneva (10). Academic community members were offered the possibility to digitize their paper vaccination records and receive a personalized assessment of their immunization status at no cost. Four to five times a year, stands were set up in different buildings of the campus. Interested students and university collaborators could bring their vaccination record, fill in a questionnaire and receive feedback on their immunization status.

An analysis of the data carried out 1 year after the launch of the project (academic year 2018–2019) revealed interesting findings. First, only 21% participants had an up-to-date immunization status, which confirmed the importance of initiatives such as the *Projet Vaccins*. Second, participants who reported having a primary care physician had no better immunization status than those who did not: only 21.5 and 20.6%, respectively, were up-to-date according to the Swiss immunization plan.

We found the latter observation puzzling because, as pointed out by others (11), checking vaccine history and catching up missing vaccination involves no medical challenge for physicians. Other kinds of barriers could play a role. For instance, we know that health care professionals have the potential of influencing patient vaccination (12) and previous studies indicate that a substantial minority of health care professionals does not systematically apply official recommendations (13–15). Therefore, one explanation for our observation may be that physicians are not convinced that all official recommendations to update vaccinations are adequate. Alternatively, physicians may forget or fail to discuss the topic with their usually healthy patients. Lack of time is a likely factor as patients often consult for acute problems (12). A further more subtle factor that has already been highlighted in the vaccine hesitancy literature may be patient's complacency (3, 16): patients may be passively resistant to checking and updating their vaccination status because they do not perceive the need to do so. Indeed, studies indicate that lack of perceived benefit from vaccination is correlated with failure to vaccinate (17). This hypothesis requires to investigate more closely adults patients' perception of their own vaccination status and own risk evaluation.

In order to investigate the matter, we decided to check whether the trend observed in 2018–2019 was confirmed in a subsequent academic year, and to examine the reasons behind it. To this end, we added new questions to the original questionnaires. Our aim was to investigate whether our participants address the topic of vaccination with their primary care physician, how they interpret and evaluate the vaccination recommendations that they receive, and what they believe about their own immunization status.

## Methods

### Main and Secondary Objectives

Our primary objective was to confirm (or reject) the hypothesis that the fact of having a primary care physician has no significant impact on the immunization status of adult patients. Thus, our primary outcomes are:

- The number of up-to-date participants among those who have a primary care physician (Group P) compared to those who do not have one (Group No-P).
- Per participant, the number of diseases for which a vaccination update is recommended (Group P compared to Group No-P).

Our second objective was to investigate in a more exploratory way the reasons behind the little impact of primary care physicians on their patients' immunization status. Many, possibly co-occurring, factors may impede physicians to update their patients' immunization status. To some extent, our data allow to check for the effect of some of these factors. Our outcomes are:

- The correlation between the frequency of medical consultations and the fact of being up-to-date. This is a proxy for the frequency of occasions to discuss the issue and make vaccination updates. A positive correlation may highlight that physicians are willing but lack time or opportunities to take care of their patients' immunization status.
- The proportion of reported discussions about vaccination with the physician in Group P. A low proportion would indicate that physicians do not include the immunization status of their patients in their routine medical examinations.
- A particularly low or high level of vaccination for specific diseases in Group P (compared to Group No-P) in contradiction with the FOPH recommendations. Such a difference would indicate that physicians do not strictly follow the official public health recommendations for their adult patients (possibly because they partially disagree with the official recommendations, or because they provide more patient-personalized immunizations).
- Participants' personal conviction to have an up-to-date immunization status and the correlation between their belief and their actual status. Overoptimistic beliefs would partially explain the lack of interest in updating own immunization status. It would highlight patients' passive resistance toward addressing the issue of vaccination.
- Participants' capacity to recall their immunization status (and the recommendations they received) after 2–3 months. Biased memories may indicate a lack of interest or a passive resistance toward immunization updating.

### Selection and Description of Participants

Participants (*n* = 432) were members of the academic community of Geneva University that counts about 17,700 students and 6,700 collaborators. Our dataset, includes 57% students; 43% university collaborators (doctoral students, researchers, teachers, administration, and technical staff); ages ranged between 18 and 76 years (median: 28, mean: 34.7); 41.6% were males and 58.4% females. Participation was voluntary and not remunerated, but vaccine digitalization was offered at no cost (sparing compared to the online costs : CHF 10.-). The recruitment took place at the *Projet Vaccins* stands. Thus, by coming to this stand, all participants showed their interest in knowing their immunization status.

### Study Design

The recruitment took place in several sessions organized by the *Projet Vaccin* (four to five times a year in various buildings on Geneva University campus). All the members of the Geneva academic community were notified (by e-mail and by a poster campaign) of the possibility to have their immunization record digitized on the Swiss electronic vaccination record platform (www.mesvaccins.ch) and to receive personalized feedback and recommendations related to their immunization status. At the stands, interested participants signed an informed consent form and answered a questionnaire before handing out their paper-based immunization record. To evaluate participants' immunization status, within a few days or weeks following the recruitment, the staff of the *Projet Vaccins* entered all the vaccines reported in participants' immunization records in www.myvaccines.ch, the secured website using the viavac software whose algorithms are based on the Swiss national vaccination recommendations (18). The system automatically outputs individualized immunization check-ups and vaccination recommendations. Participants received an e-mail notification informing them about their status (“You are up-to-date” or “You are not up-to-date”) and the recommendation to log on their newly created online account on the Swiss electronic vaccination record platform (www.myvaccines.ch), which provides personalized vaccination information. Log in to the platform was made easy thanks to a separate e-mail recalling participants' ID. Myvaccines platform provides vaccination recommendations (green and red lights) specifically designed for being understandable by the general population. Approximately 3 months later, participants were contacted again by e-mail and asked to answer a second online questionnaire.

During the academic year 2018–2019, participants were asked to answer a limited number of questions (Supplementary File 1). Notably, in the recruitment questionnaire, we asked participants whether they have a primary care physician, and how frequently they visit their physician. In the second questionnaire, we asked them whether they are up-to-date, and if not, whether they remember what vaccinations was recommended to them.

Based on these preliminary results, we decided to investigate further the role of primary care physicians as well as participants' beliefs about their vaccination status. We therefore added more questions during the second year 2019–2020. To investigate more directly whether primary care physicians addressed vaccination issues with their patients, we asked participants whether their physician has recently checked their vaccination coverage. Moreover, assuming that people are likely to show a passive resistance toward vaccination update when they do not feel concerned, we investigated whether our participants had an accurate belief about their own vaccination status by asking them at the recruitment stage, to predict their immunization status with the possible answers “up-to-date,” “not up-to-date,” “I do not know.”

### Statistics

Data processing and analysis were done with the softwares R (version 3.6.1) and Stata/IC (Version 15.1). For group comparisons with binary data, we used the X-squared test (or the Fisher's Exact for data containing one cell below 5). For group comparisons with continuous data, we used the *t*-test. For the correlation test involving continuous variables (e.g., number of medical consultations), we used a simple linear regression model (ordinary least squares model).

To ensure that the number of participants in our study was sufficient to test the effect of having a primary care physician on immunization status, we made a *post-hoc* power proportion test (*n* = 432; sig level = 0.05; power = 0.8). The result indicates that an effect size of about 0.12 (i.e., difference in immunization status between Group P and Group No-P) would have been detected as significant with the collected sample size.

### Ethics

We submitted the research protocol to the Geneva Commission Cantonale d'Ethique de la Recherche (Project-ID: 2019-01359) who decided that the project does not fall within the scope of the Human Research Ordinance and therefore alleviated the need for ethical approval (decision date: 29.08.2019). Our research was conducted in accordance with the Helsinki Declaration. Collected data included identifiable data (e.g., name, date of birth, gender, e-mail address) and personal data (e.g., immunization status, number of recent medical consultations, opinion on own vaccination status). During the data collection phase, the research staff of the *Projet Vaccins* was bound by a confidentiality agreement. We anonymised the data before analyzing the results.

## Results

We collected data from 434 participants: 285 during the 2018–2019 academic year (with use of the original shorter questionnaires), and 149 during the 2019–2020 academic year (with the improved questionnaires). They all responded to a first questionnaire and provided their paper immunization record. Of these participants, 214 (49.3%) responded to the second online questionnaire 2–3 months, a high response rate for such surveys. In both questionnaires, some participants did not answer all questions. Two participants (one in 2018–2019 and one in 2019–2020) did not report whether they had a primary care physician and were excluded from most analyses. Detailed data are available in Supplementary File 2.

**Figure 1:**
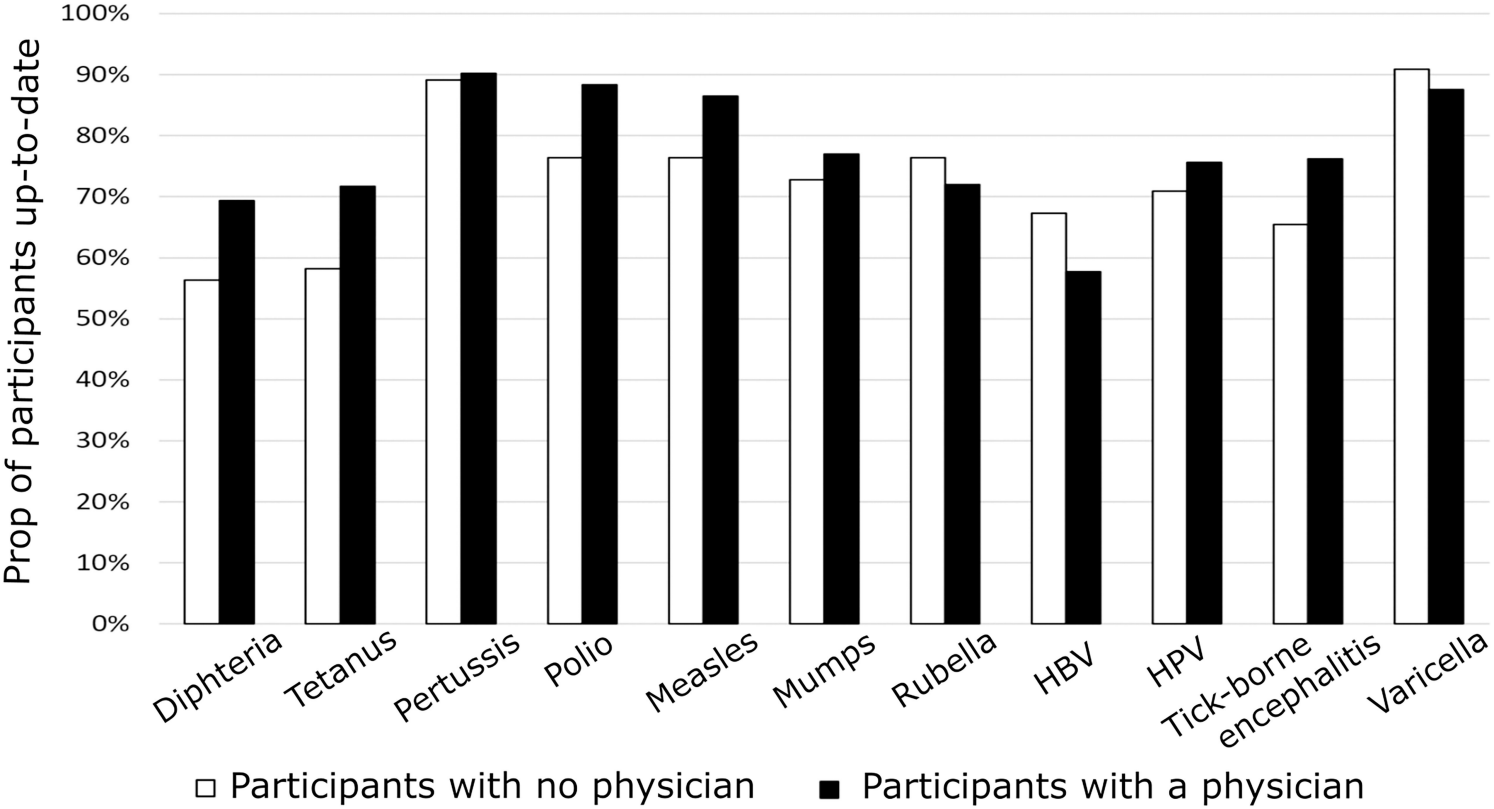
Proportion of participants (*n* = 432) that have been vaccinated according to the Swiss national vaccination recommendations. Details are provided for each targeted disease. Black bars represent participants who report having a primary care physician and white bars represent those who report having no primary care physician.

Only, 22.1 % participants were up-to-date with their immunization status. The detailed result stratified by disease (measles, rubella, etc.,) and by groups (participants reporting having, or not, a primary care physician) is illustrated in Figure 1.

Data pulled together (*n* = 432) and separated year by year (n2018–2019 = 284; n2019–2020 = 148) confirmed that the fact of having a primary care physician has little impact on the immunization status of adult patients (Figure 1 and Supplementary Table 1). Participants who reported having a physician had no better immunization status than patients who did not have a physician: respectively, 22.5 patients with physicians (21.6% in 2018–2019; 24.4% in 2019–2020) and 20% patients without physicians (20.6% in 2018–2019; 19% in 2019–2020) were up-to-date according to FOPH's 2020 recommendations.

A closer look at the number of diseases for which a vaccination update is recommended to participants confirmed that having a primary care physician does not affect the immunization status. Indeed, participants who reported having a primary care physician were insufficiently protected against 2.1 diseases, compared to 2.6 among participants without a physician. This difference was not significant (*n* = 432; *t*-test, *p* = 0.07) and clinically irrelevant.

**Figure 2:**
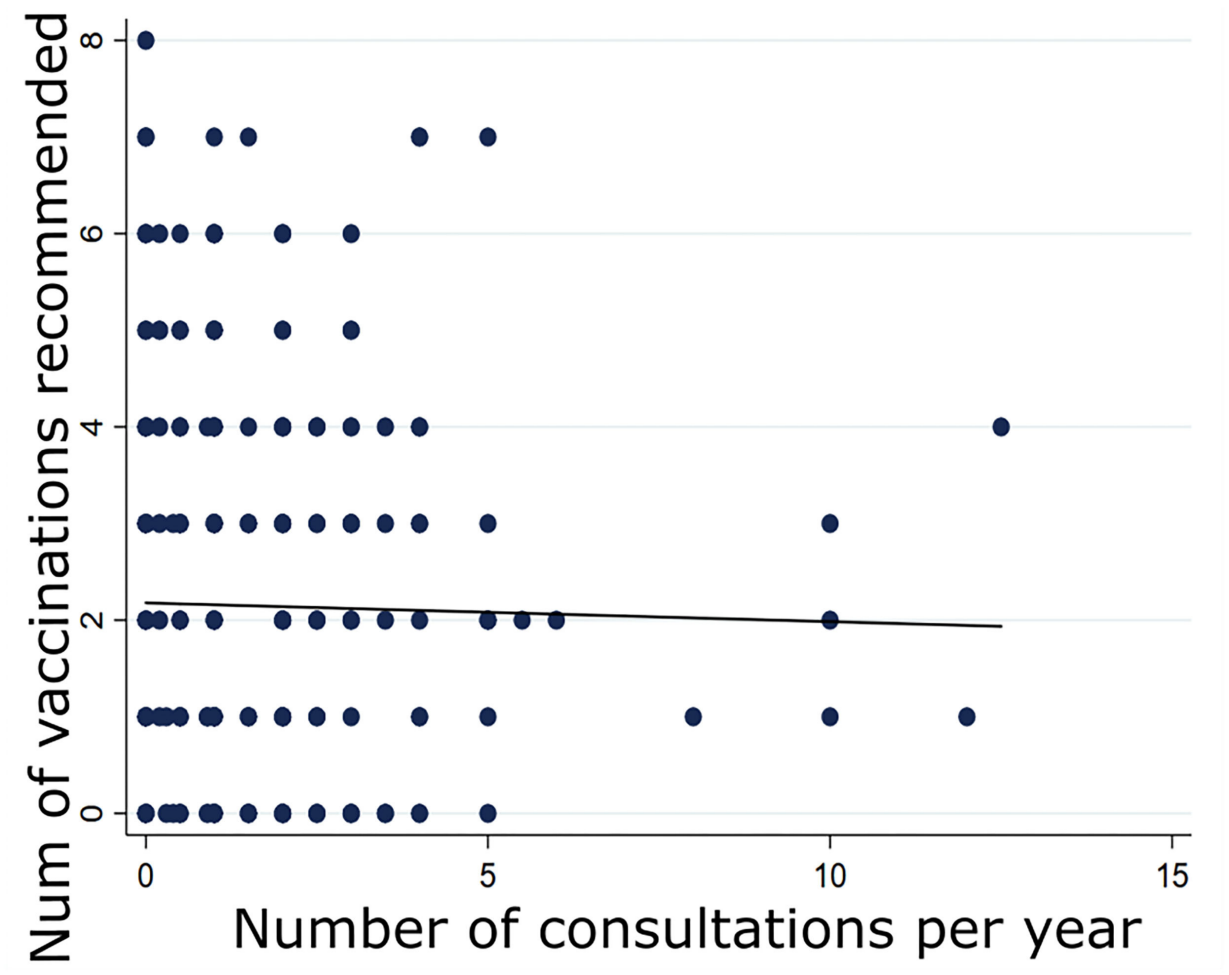
Correlation between the number of vaccinations recommended to participants and the frequency of their medical consultations (*n* = 431).

To check whether patients that consulted more often (and thus had more occasions to discuss vaccination issues with their physician) were more likely to be up-to-date with their immunization status, we assessed whether the number of vaccines recommended correlated with the frequency of appointments (Figure 2). For this analysis, we excluded one participant who reported a high (>24) number of annual consultations that does not represent the health status of mostly healthy university students and collaborators. A simple linear regression analysis (*n* = 431; coefficient = −0.019; SD = 0.056; *p* > 0.1) indicated that the frequency of medical consultations did not impact on our participants' immunization status.

To investigate more directly whether primary care physicians addressed vaccination issues with their adult patients, we assessed the percentage of Group P participants reporting that their physician has recently checked their vaccination coverage (only 2019–2020 data are available for this test; *n* = 127; Supplementary Table 1). Thirty-seven participants (29.1%) reported that this was the case; among them, only 16.2% were up-to-date, compared to 27.5% among participants that did not show their vaccination record to their physician (this sample size is small and the difference is not statistically significant: X-squared test, *p* > 0.1).

As illustrated in Figure 1, we did not find evidences that physicians' assessment of which vaccines need (or not) to be updated, mismatches with the official FOPH recommendations: patterns of vaccination were not significantly different between Group P and Group No-P, indicating that physicians are not particularly reluctant (or prone) to update specific vaccines.

Among participants who made a prediction about their immunization status (only 2019-2020 data are available for this test; *n* = 97; Supplementary Table 2), 61.5% made a wrong prediction. More specifically, among participants who predicted that they were “up-to-date,” 71.2% were diagnosed as “not up-to-date” (= wrong belief), whereas among participants who predicted that they were “not up-to-date,” only 11.8% expressed a wrong belief (Fisher's exact test, *p* <0.001).

When asked to report whether they had received a recommendation to update their immunization status or the assessment that they were up-to-date, most participants (*n* = 134/149, 90%) reported that they did remember the information they received (Supplementary Table 3). The error rate of this remembrance was significantly greater (X-squared test, *p* < 0.001) among participants who remembered that they were up-to-date (52.8%) than among participants who remembered the reverse (6.2%). Overall, this result indicates that people who believe that they are up-to-date tend to forget or misunderstand contrary official information provided on their immunization status.

## Discussion

All our results converge towards the conclusion that primary care physicians do not contribute significantly to their adult patients' immunization status. Even patients who regularly meet their physician, or who report having checked their vaccination status with their physician were not better immunized. Various explanations may be offered for this lack of impact, and we provide preliminary results to evaluate their robustness.

Our data do not support the hypothesis that physicians disagree with the FOPH recommendations: none of the recommended vaccines seemed to be particularly promoted or prevented in group P. This is good news from a public health point of view.

One possible explanation for our observations is that physicians simply fail to address the topic with their adult patients. Indeed, even though most of our participants were interested in updating their vaccination records (they freely entered in our study for this purpose), only 28% in group P reported having recently checked their vaccination coverage with their physician.

A series of (co-occuring) factors may explain the physicians' observable lack of contribution to their patients' immunization status. Insufficient time or opportunities to address the topic (and make the necessary updates) is a likely factor, although, this hypothesis is not backed up by our data: in our sample, patients that consulted their physician more often showed no better immunization status. Practical barriers such as vaccine storage limitations may also come in the way. Our data do not help to assess these explanatory factors.

Our results however highlight the possible impact of a further more subtle factor: adult patients, and possibly their physicians as well, may passively resist to checking and updating vaccination status because they do not perceive the need to do so. We indeed found a strikingly high rate of participants' wrong predictions about their immunization status, and high rate of forgetting or misunderstanding official information provided on their immunization status. The rate of wrong beliefs is particularly high among patients that are optimistic about their status, which indicates the presence of a perceptual bias: a tendency to be overconfident about their status (19, 20). This bias is possibly due to a lack of awareness of the risks posed by some diseases that have become uncommon (7). It may also be linked to the belief that vaccination is only relevant for children (21). More prosaically, it may simply express a feature of the human mind. To illustrate, data recently collected during the COVID-19 pandemic show that people are more worried about the potential health impact of the virus on others than on themselves (22).

In conclusion, the pattern of beliefs that we observe in our study indicates that our participants were unworried about their own immunization status because they held overoptimistic beliefs about their protection. This result is particularly interesting in light of the existing literature on complacency (16). It highlights not only that participants perceive risks of vaccine-preventable diseases as low, if they are note vaccinated. In addition, they tend to think that they are immunized against these diseases. Such a phenomenon may reflect humans' tendency to post-rationalize (23) and avoid cognitive dissonance (24). This result highlights the important causal power of complacency which is sometimes overlooked in research programs mainly focused on (also relevant) explanatory factors related to population's concerns about the lack of efficacy or adverse effects of vaccination (25). Patients in our study failed to update their immunization status although they did not express such concerns.

These data need to be confirmed with a larger and more balanced sample size. Indeed, our data are limited in several respects because we did not sample randomly from the general population: all participants are members of the academic community in Geneva and most of them are young. All participants found it relevant to check their immunization status and voluntarily came to the *Projet Vaccins*' stands. Our participants are thus likely to be favorably disposed toward vaccination. Consequently, the rate of participants' refusal to update their vaccination status (11.4% in our study) is probably an underestimation, and reversely, the rate of individuals who have their vaccination record checked by their physician (28% in our study) may be an overestimation.

Except for our main outcome (physicians' lack of impact), our results need to be considered as exploratory because we tested multiple hypotheses with one single dataset. Based on our study, it would be interesting to further confirm people's overconfidence in their immunization status and to investigate more in detail the reasons underlying these wrong beliefs. It would also be interesting to study physicians' point of view on the matter. Do physicians check vaccination records within their routine medical examinations of adults or only when these patients plan to travel to foreign countries? How do physicians estimate the risk of a poor immunization status for their adult patients, compared to the risk for children? What barriers do physicians encounter when they decide to update the immunization status of their patients? These questions related to immunization updating have been somewhat neglected compared to research on children or on first vaccination (21).

Despite its limitations, our study makes it clear that more efforts are needed in order to motivate or to help physicians to address the issue of vaccination with their adult patients, and to convince them to update their vaccines. Interestingly, the COVID-19 crisis may provide a rare opportunity to primary care physicians. Numerous patients will ask for a coronavirus vaccination. Most of these patients do not realize that they are insufficiently immunized against illnesses that are even more serious. We urge primary health physicians to take the opportunity to check the overall status of their patients and make the necessary updates.

## Data Availability Statement

The original contributions presented in the study are included in the article and Supplementary File 2. Further inquiries can be directed to the corresponding author/s.

## Ethics Statement

Upon submission of the protocol, the local research ethics committee decided that ethical review and approval was not required for the study on human participants in accordance with the local legislation and institutional requirements. The patients/participants provided their written informed consent to participate in this study.

## Author Contributions

TP and CC contributed to the conception and design of the study, to the data analysis and interpretation, and to the article drafting. TP was in charge of the data collection. All authors contributed to the article and approved the submitted version.

## Conflict of Interest

The authors declare that the research was conducted in the absence of any commercial or financial relationships that could be construed as a potential conflict of interest.
