# Supplementary material for: Do Primary Care Physicians Contribute to the Immunization Status of Their Adult Patients? A Story of Patients' Overconfidence Coupled With Physicians' Passivity
Source: Front Med (Lausanne). 2021 Jun 17;8:655734. doi: 10.3389/fmed.2021.655734 (PMC8245703; doi:10.3389/fmed.2021.655734)
Supplement: Supplementary file 1 [file Table_1.DOCX]

Supplementary File 1 – Questionnaires

| **Questions, discussed in the article, included in questionnaire 1 (at recruitment)** | |
| --- | --- |
| french (original) | english translation |
| Avez-vous un médecin traitant / médecin généraliste ? [Oui / Non]  si oui, fréquence approximative de visite [x/an] | Do you have a primary care physician? [Yes / No]  if yes, approximate frequency of visit [x/year] |
| Additional questions to questionnaire 1 that were used in 2019-2020 | |
| Est-ce que votre médecin traitant habituel s’est intéressé à votre couverture vaccinale ? A-t-il récemment regardé votre carnet de vaccination ? [Oui (quand?) / Non]  Si vous deviez prédire votre statut vaccinal, vous diriez que vous êtes… ? [A jour / Non à jour / Je ne sais pas] | Did your primary care physician show interest in your immunization status? Has he or she recently looked at your vaccination record? [Yes (when?) / No]  If you had to predict your immunization status, you would say that you are…? [Up-to-date / Not up-to-date / Don't know] |
| **Questions, discussed in the article, included in questionnaire 2 (2-3 months after recruitment)** | |
| Quand vous avez vu vos résultats dans le bilan vaccinal du site mesvaccins.ch, aviez-vous des vaccins non à jour (lumières rouges)? [Oui / Non / Je ne me souviens plus / je ne suis pas retourné sur mon compte mesvaccins.ch depuis son ouverture]  Y a-t-il des vaccins parmi ceux qui vous sont recommandés que vous ne souhaitez pas faire? [Oui / Non]  si oui lesquels? [liste à cocher des vaccins possibles]  si oui, pour quelle raison ne voudriez-vous pas faire ce(s) vaccins(s)? [boîte de texte] | When you saw your results in the vaccination check-up on the mesvaccins.ch website, did you have any vaccines that were not up-to-date (red lights)? [Yes / No / I don't remember / I haven't returned to my mesvaccins.ch account since it was opened]  Are there any vaccinations among those recommended that you do not want to update? [Yes / No]  If yes, which ones? [checklist of possible vaccines]  If yes, why would you not want to update those vaccine(s)? [text box] |
