# Supplementary material for: Do Primary Care Physicians Contribute to the Immunization Status of Their Adult Patients? A Story of Patients' Overconfidence Coupled With Physicians' Passivity
Source: Front Med (Lausanne). 2021 Jun 17;8:655734. doi: 10.3389/fmed.2021.655734 (PMC8245703; doi:10.3389/fmed.2021.655734)
Supplement: Supplementary file 2 [file Table_2.DOCX]

Supplementary File 2 – Summary tables of results

|  | Group P | Group No-P |
| --- | --- | --- |
| 2018-2019 : total nb of participants = 284 | | |
| Up-to-date | 54 | 7 |
| Not up-to-date | 196 | 27 |
| 2019-2020: total nb of participants = 148 | | |
| Up-to-date | 31   \| Record-Checked \| Record-Not-Checked \| \| --- \| --- \| \| 6 \| 25 \| | 4 |
| Not up-to-date | 96   \| Record-Checked \| Record-Not-Checked \| \| --- \| --- \| \| 31 \| 65 \| | 17 |

Table 1. Number of participants reporting having (Group P) or not having (Group No-P) a primary care physician that are up-to-date (or not) with their immunization status. Among participants from the academic year 2019-2020, number of participants that report having (or not) checked their vaccination record with their physician.

|  | **Up-to-date** | **Not up-to-date** |
| --- | --- | --- |
| **Predict being up-to-date** | 23 (= correct belief) | 57 (= wrong belief) |
| **Predict being not up-to-date** | 2 (= wrong belief) | 15 (= correct belief) |
| **Do not know** | 10 | 43 |

Table 2. Among participants from the academic year 2019-2020, number of participants that predict having (or not) an up-to-date immunization status at recruitment time.

|  | **Received Up-to-date info** | **Receive Not up-to-date info** |
| --- | --- | --- |
| **Remember being up-to-date** | 25 (= correct belief) | 28 (= wrong belief) |
| **Remember being not up-to-date** | 5 (= wrong belief) | 76 (= correct belief) |
| **Do not remember or did not check the information** | 3 | 12 |

Table 3. Number of participants that remember having received the information that they were up-to-date (or not, or do not remember) 2-3 months after having received their immunization status.
